# Supplementary material for: Increased phagocytosis in the presence of enhanced M2-like macrophage responses correlates with increased primary and latent HSV-1 infection
Source: PLoS Pathog. 2020 Oct 8;16(10):e1008971. doi: 10.1371/journal.ppat.1008971 (PMC7575112; doi:10.1371/journal.ppat.1008971)
Supplement: Attachment — Submitted filename: Response (08-27-2020)PP.doc [file ppat.1008971.s001.doc]

**RE**: PPATHOGENS-D-20-01670 (Increased phagocytosis in the presence of enhanced M2-like macrophage responses correlates with increased primary and latent HSV-1 infection)

Dear Drs. Jones, Flemington, Haldar and Malim:

Below are our detailed responses to each of the reviewers' comments, indicating the changes we have made to the manuscript. Thank you for the opportunity to revise and resubmit our manuscript.

**REVIEWER #1:**

Summary: Previous work from the Ghiasi laboratory has shown that macrophages of the M2 phenotype are more protective than macrophages of the M1 phenotype against HSV-1 primary corneal infection and latency. Ghiasi and coworkers continued their investigations of M1 vs M2 macrophage phenotypes on the pathogenesis of HSV-1 corneal disease in the present investigation through development and use of novel C57BL/6 mice that either lack M2 macrophages (M2-/- mice) or overexpress M2 macrophages (M2-OE). This investigation examined further the impact of a deficiency in M2 macrophages as well as the impact of overexpression of M2 macrophages on HSV-1 corneal disease and latency at the ganglionic level with some emphasis on the phagocytotic activity of macrophages that infiltrate the cornea following primary HSV-1 corneal infection. Analysis of several experimental endpoints appropriate for a study of HSV-1 corneal infection and latency revealed no effect on phagocytosis, virus replication as measured in tear films for infectious virus, ganglionic latency, and cytokine expression when M2-/- mice were compared with wildtype mice. In contrast, overexpression of M2 macrophages in M2-OE mice when compared with wildtype mice revealed increased phagocytosis, increased virus replication at the corneal surface during primary infection, increase ganglionic latency, and increased expression of cytokines, both pro-inflammatory and anti-inflammatory. The authors conclude that the proper balance of M1 vs M2 macrophage phenotypes is important in controlling HSV-1 corneal disease.

Review: This is a well written manuscript that summarizes the findings of an extension of work previously published by the Ghiasi laboratory on the role of M1 vs M2 macrophages during the pathogenesis of primary HSV-1 corneal infection, ganglionic latency, and ganglionic reactivation. Little is known about macrophage phenotypes and HSV-1 disease pathogenesis, so this investigation is a worthwhile pursuit. Of greater significance, the study uses two mouse strains unique to the Ghiasi laboratory. The studies are carried out carefully with appropriate use of statistical analysis. While no major concerns are apparent, attention to a few minor issues would serve to strengthen the manuscript.

**Minor point 1**. There is no discussion of so-called “M0” macrophages. Do they exist in the M2-/- and M2-OE mice? If so, to what extent and to what end pathogenetically?

*RESPONSE*: M0 are untreated macrophages and exist in both M2^-/-^ and M2-OE mice and after polarization generate M1 and M2 types. They play no role in pathogenicity. The following sentence is now added to the revised manuscript…. “Both M1 and M2 macrophages are generated from untreated macrophages (M0).” (Lines 70-71)

**Minor point 2**. Peritoneal macrophages are collected from wildtype, M2-/-, and M2-OE mice for initial comparisons. While the findings are convincing and compelling, do the peritoneal macrophage populations faithfully represent the macrophage populations of the cornea during primary HSV-1 infection?

*RESPONSE*: Macrophage population is tissue specific and peritoneal macrophages may not be similar to that of the macrophage population in the cornea.

**Minor point 3**. Some clarification is needed regarding the precise role of macrophages during primary HSV-1 infection of the corneal surface. It is understood that (1) macrophages are recruited to the corneal surface during primary HSV-1 infection, and (2) macrophages function has phagocytes during primary HSV-1 infection. The authors then conduct an experiment to compare peritoneal macrophages from wildtype mice, M2-/- mice, and M2-OE mice for HSV-1 replication. Why? Do the authors believe that the infiltrating macrophages, in addition to serving as phagocytes, also serve as a target for virus infection and replication and thereby increase the amount of virus found in tear films? If so, what percentage of infiltrating macrophages become infected during the course of primary HSV-1 infection

*RESPONSE*: Previously we have shown that macrophages are efficiently infected with HSV-1 but they do not efficiently support virus replication. This study was performed to determine if macrophages from M2^-/-^ and/or M2-OE mice support virus replication more efficiently than WT mice. Thus, the following two statements are now added to the revised manuscript and read as follow….”Previously we have shown that macrophages from WT mice after polarization towards M1 state had lower viral replication in comparison to macrophages polarized towards M2 state (Lines 148-150).” “Although we added the following….. “Our current study supports our previous studies with regards to higher virus titer in M2-OE mice as compared to WT or M2^-/-^ mice.” (Lines 164-165).

**Minor point 4**. The subheading within the Results section of Lines 177 and 176 is awkward and needs revision.

*RESPONSE*: Agree. This subheading is now changed to…”Role of M2 macrophages on survival and eye disease in infected mice”.

**REVIEWER #2:**

This is an interesting manuscript that focuses on describing the significance of M1/M2 macrophages in HSV-1 primary and latent infections. Until now very little has been known on their specific roles in HSV-1 infection. To address the significance the authors generated M2-/-mice as well as conditional transgenic mice overexpressing M2 macrophages. They found clear evidence that higher expression of M2 macrophages correlated with higher phagocytosis, as well as higher HSV-1 replication. The latter led to an increase in latency. At the cytokine level they also noticed an increase in IL-4 and IFN-γ expression. Quite interestingly, they found that the lack of M2 macrophages did not affect HSV-1 infectivity. Manuscript is well written, provides highly significant information and easy to follow. The main conclusion that a balanced proportion of M1/M2 macrophage may be an effective way to control the infection is well supported by the results shown in the manuscript.

**Minor issue 1**. Under introduction please add a couple of sentences on HSV-1 ocular pathogenesis. Please also comment whether HSV-1 is known to cause primary infection in the cornea.
*RESPONSE*: The following sentence is now added and reads as follow… “In immunopathological diseases like herpes stromal keratitis (HSK), both T cells and innate immune cells play a major role in combating the virus replication which could eventually lead to HSK if not properly controlled”. (Lines 53-55).

**Minor issue 2**. Line 104, please explain the functional need for Rosa26.

*RESPONSE*: The following sentence was added to M&M section and read as follow….”Rosa26 mouse line was used to overcome challenges associated with macrophage permeabilization and quantification”. (Lines 330-331).

**Minor issue 3**. Line 212, please cite a reference for exhaustion markers used in the study.
*RESPONSE*: Agree. A reference is now added to line 212.

**Minor issue 4**. Line 336, please add the original source for viral strains used in the study.
*RESPONSE*: Agree. The original source of virus is now added.

**Minor issue 5**. Fig. 1, please expand HE and HO in the figure legend.
*RESPONSE*: Done (Lines 639-640).

**Minor issue 6**. Fig. 2, this and other figures please uniformly use either WT (listed in the legend) or control (listed in the figure).

*RESPONSE*: Agree. All control were changed to WT.

**REVIEWER #3:**

This is an excellent study from Dr. Ghiasi’s group. In this study, the authors showed that over expression but not lack of M2 macrophage population increases the load of HSV-1 replication and latency in infected mice. However, over expression of M2 population does not affect HSV-1 reactivation. The conclusions made were strongly supported by the data presented in the manuscript. Strength of the study involve the use of M2 knockout and M2 over expressing mice in studying the role of M2 macrophage population in HSV-1 infectivity, latency and corneal disease. Overall, a well thought study delineating the role of M2 macrophage population in the mouse model of corneal HSV-1 infection.

**Minor issue 1**. In methods, please provide details of how normalized fold expression was calculated.
*RESPONSE*: The following was added to the revised manuscript….”By comparing the normalized threshold cycle (C_T_) of each sample to the threshold cycle of the standard curve, the copy number for each reaction product was determined. For fold change of expression analysis, the 2^−ΔΔCT^ method was used to calculate gene expression fold change compared to expression in uninfected controls. (Lines 427-431).

**Minor issue 2**. In Figure 6, did authors measure the protein level of IL-4 and IFN-g in the corneas of infected mice? Please comment.

*RESPONSE*: This is an interesting idea and we plan to look at protein expression in cornea of infected mice in our future studies.

**Minor issue 3**. In Figure 9, the author showed higher level of PD-1 in TG of M2 over expressing mice. What cell population is expressing higher level of PD-1 in the latent TG?
*RESPONSE*: PD-1 is mainly expressed by T cells. We did not perform FACS on isolated TG in our current study.

**Minor issue 4**. In the discussion, can authors discuss the translational implications of their results

*RESPONSE*: The following sentences are now added at the end of discussion and read as follow…”A barrier to the effective alleviation of the public health burden of HSV-1-induced corneal damage and blindness has been the identification and efficient targeting of the main immune responses. Our study suggest that a balance of M1 and M2 phenotype will reduce ocular viral load and reduce corneal damage and latency-reactivation”. (Lines 310-313).

Thank you very much for allowing us to revise and resubmit our manuscript. We trust that we have satisfactorily interpreted these helpful reviewers’ comments, and we have now made all the changes that were requested by the reviewers. New text in the body of the manuscript is printed in “Blue Arial” rather than “Black Arial”. We hope that the manuscript will now be found to be acceptable for publication in PLOS Pathogen.

Best Regards,

Homayon Ghiasi
